# Disruption of Extracellular Signal-Regulated Kinase Partially Mediates Neonatal Isoflurane Anesthesia-Induced Changes in Dendritic Spines and Cognitive Function in Juvenile Mice

**DOI:** 10.3390/ijms26030981

**Published:** 2025-01-24

**Authors:** Swati Agarwal, Jacqueline Bochkova, Mazen K. Mohamed, Michele L. Schaefer, Annika Zhou, John Skinner, Roger A. Johns

**Affiliations:** 1 Department of Anesthesiology and Critical Care Medicine, Johns Hopkins University, Baltimore, MD 21205, USA; sagarw33@jhmi.edu (S.A.); michele.schaefer@qiagen.com (M.L.S.); jskinne8@jhmi.edu (J.S.); 2 Krieger School of Arts and Sciences, Johns Hopkins University, Baltimore, MD 21218, USA; jacquelineb1@gwmail.gwu.edu (J.B.); mam1667@pitt.edu (M.K.M.); zhouannika17@gmail.com (A.Z.)

**Keywords:** isoflurane, dendritic spines, hippocampus, cognition, ERK, pizotifen

## Abstract

There is a growing concern worldwide about the potential harmful effects of anesthesia on brain development, based on studies in both humans and animals. In infants, repeated anesthesia exposure is linked to learning disabilities and attention disorders. Similarly, laboratory studies in mice show that neonates exposed to general anesthesia experience long-term cognitive and behavioral impairments. Inhaled anesthetics affect the postsynaptic density (PSD)-95, discs large homolog, and zona occludens-1 (PDZ) domains. The disruption of the synaptic PSD95-PDZ2 domain-mediated protein interactions leads to a loss of spine plasticity and cognitive deficits in juvenile mice. The nitric oxide-mediated protein kinase-G signaling pathway enhances synaptic plasticity also by activating extracellular signal-regulated kinase, which subsequently phosphorylates cAMP-response element binding protein, a crucial transcription factor for memory formation. Exposure to isoflurane or postsynaptic density-95-PDZ2-wildtype peptides results in decreased levels of phosphorylated extracellular signal-regulated kinase (p-ERK) and phosphorylated cAMP-response element binding protein (p-CREB), which are critical for synaptic plasticity and memory formation. Pizotifen treatment after isoflurane or postsynaptic density-95-PDZ2-wildtype peptide exposure in mice prevented decline in p-ERK levels, preserved learning and memory functions at 5 weeks of age, and maintained mushroom spine density at 7 weeks of age. Protein kinase-G activation by components of the nitric oxide signaling pathway leads to the stabilization of dendritic spines and synaptic connections. Concurrently, the ERK/CREB pathway, which is crucial for synaptic plasticity and memory consolidation, is supported and maintained by pizotifen, thereby preventing cognitive deficits caused in response to isoflurane or postsynaptic density-95-PDZ2-wildtype peptide exposure. Activation of ERK signaling cascade by pizotifen helps to prevent cognitive impairment and spine loss in response to postsynaptic density-95-PDZ2 domain disruption.

## 1. Introduction

There are numerous unanswered questions about developmental anesthesia neurotoxicity despite a wealth of research within pediatric perioperative care [1]. Laboratory animal research, particularly studies involving nonhuman primates, has shown that early exposure to anesthetics can lead to long-term cognitive dysfunction and impaired neurocognitive performance [2,3,4]. However, the findings from clinical studies do not fully align with those from laboratory animal studies due to differences in experimental designs and outcome measures used in laboratory versus clinical investigations [1,5,6]. Recent advances in the field of developmental anesthesia neurotoxicity have demonstrated that anesthetic exposure can result in deficits in specific neurocognitive domains [1,7]. Despite these advances, the underlying mechanisms of these neurological deficits remain incompletely understood. It is, therefore, essential to elucidate the cellular and molecular mechanisms impacted by anesthetic exposures that lead to adverse neurodevelopmental outcomes.

Ion channels and receptors are prime targets of general anesthetics, which are linked with their downstream signaling components through PDZ domain-mediated protein–protein interactions [8,9,10,11,12,13]. Our previous studies have shown that inhaled anesthetics interfere with PDZ domain-mediated protein–protein interactions at membrane receptors involved in neuronal excitation and pain processing [12,13]. Furthermore, clinically relevant concentrations of inhalational anesthetics block PDZ2 domain-mediated protein–protein interactions between postsynaptic density (PSD) proteins (such as PSD-95 or PSD-93) and N-methyl-D-aspartate receptor (NMDAR) or neuronal nitric oxide synthase (nNOS) in a dose-dependent manner [12,14,15]. We have utilized active PSD-95 wild-type PDZ2 peptides, which disrupt postsynaptic density PDZ2-mediated protein interactions by binding to interaction partners [12,13]. This disruption significantly reduced the minimum alveolar anesthetic concentration and righting reflex EC50, indicating that this domain and protein are crucial for anesthetic action.

PSD-95 PDZ2 interaction with the NMDA receptor promotes the plasticity of excitatory synapses [16]. Notably, our previous studies demonstrated that clinically relevant concentrations of isoflurane disrupt the PDZ2 domain-mediated protein interaction between NR2A/2B and PSD-95 in the hippocampus of neonatal mouse brains, impacting underlying mechanisms in hippocampal neurons [13]. In earlier investigations, we examined the effects of isoflurane and PSD-95 wild-type PDZ2 or PDZ2WT peptide on dendritic spines and synapses, which are vital for brain plasticity. We identified novel and potentially complex interactions between nitric oxide (NO) and downstream effector molecules [13,17]. Activation of protein kinase-G by components of the NO signaling pathway has been shown to prevent the loss of early dendritic spines and synapses in neurons, mitigating cognitive impairment in mice following disruption of the PDZ2 domain of the PSD-95 protein [13]. The NO-cGMP-PKG signaling pathway coordinates the regulation of ERK and ERK-driven gene expression at pre- and postsynaptic sites [18].

In this study, we investigated the impact of isoflurane exposure or synaptic PSD-95-PDZ2 disruption on subsequent long-term memory and spine plasticity in both male and female mice, as well as the perturbation of ERK signaling. We employed pizotifen as a pharmacological activator of the ERK pathway to determine if injecting it in neonatal mice could prevent the loss of protein kinase-G signaling induced by isoflurane or PSD-95 wild-type PDZ2 peptides, thereby preserving spine plasticity and cognitive function. Additionally, we tested the effect of pizotifen in neonatal mouse pups to see if it could prevent the decrease in phosphorylation of ERK or CREB in response to synaptic PSD95-PDZ2 domain disruption. Pizotifen was introduced in mice at the time of isoflurane anesthesia or PSD95-PDZ2 wild-type peptide administration to evaluate its potential in preventing cognitive impairments at 5 weeks of age and loss of mushroom spine density at 7 weeks of age in both male and female mice.

## 2. Results

### 2.1. Pizotifen Activated ERK Pathway in Neonatal Isoflurane- or PDZ2WT Peptide-Exposed Mice

**Figure 1:**
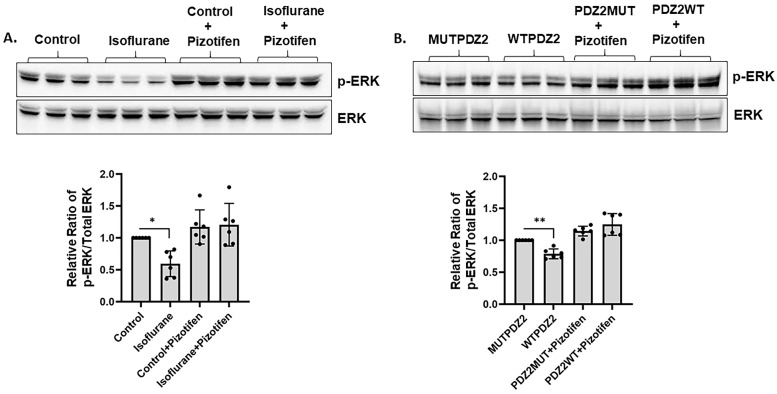
Pizotifen increases p-ERK levels in neonatal isoflurane or PDZ2WT peptide-exposed mice. At postnatal day 7, mouse brains were harvested after exposure to the following conditions: control (50% oxygen: control), 1.5% isoflurane in 50% O_2_ (isoflurane), control condition + pizotifen (10 mg/kg, ip), isoflurane + pizotifen, 8 mg/kg inactive PSD-95 mutant PDZ2 peptide (referred to as PDZ2MUT), 8 mg/kg active PSD-95 wild-type PDZ2 peptide (referred to as PDZ2WT), PDZ2MUT + pizotifen, and PDZ2WT + pizotifen. (**A**) Representative Western blot showing levels of phospho-ERK in crude hippocampal lysate in response to gas exposures in the presence or absence of 30 min treatment with pizotifen. Lower panel shows the densitometric analysis of p-ERK/Total ERK levels. (**B**) Representative Western blot showing levels of phospho-ERK in response to peptide in the presence or absence of pizotifen. Lower panel shows the densitometric analysis of p-ERK/Total ERK levels. Data were analyzed using 1-way ANOVA with Bonferroni’s multiple comparisons test. Data represent mean ± SD (n = 6 mice per group). ** *p* < 0.01 and * *p* < 0.05 compared to control or PDZ2MUT or as indicated.

To investigate the downstream signaling events and mechanisms of action, we measured the effect of isoflurane or PDZ2WT peptides in PND7 mice pups on ERK phosphorylation. Through Western blotting, we found a significant decrease in the phosphorylation of ERK in the hippocampal lysate in response to isoflurane (Figure 1A) or PDZ2WT peptide (Figure 1B) exposure. However, injecting neonatal pups with 10 mg/Kg pizotifen for 30 min at the time of exposure prevented the decrease in ERK phosphorylation that occurred after isoflurane and PDZ2WT peptide exposure. Thus, pizotifen treatment prevented the loss of phospho-ERK levels in the hippocampal lysates extracted from neonatal mice brains (Figure 1A,B). The data were analyzed using one-way ANOVA, which revealed that the levels of phosphorylated ERK relative to total ERK were significantly decreased in both the isoflurane group compared to the control (*p* = 0.0149) and in the PDZ2WT peptide group compared to the PDZ2MUT peptide group (*p* = 0.0031). No significant differences were observed between control + pizotifen and isoflurane + pizotifen (*p* > 0.999) or between PDZ2MUT + pizotifen and PDZ2WT + pizotifen (*p* = 0.1742). Six mice per group were used in the analysis.

### 2.2. Neonatal Isoflurane Exposure Caused Decrease in CREB Phosphorylation Prevented by Pizotifen Injection

**Figure 2:**
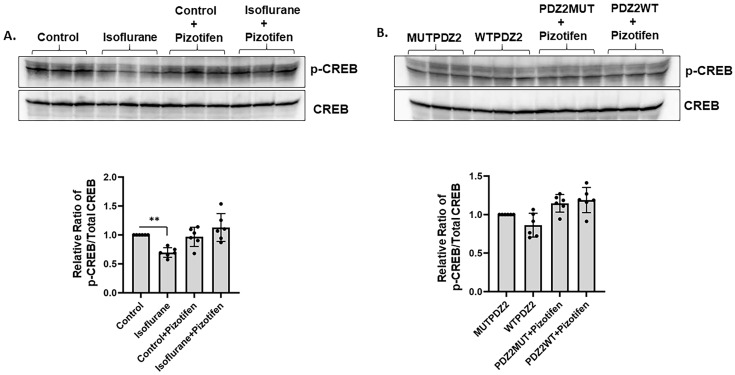
Neonatal isoflurane exposure caused a decrease in CREB phosphorylation prevented by pizotifen injection. At postnatal day 7, mouse brains were harvested after exposure to the following conditions: control (50% oxygen: control), 1.5% isoflurane in 50% O_2_ (isoflurane), control condition + pizotifen (10 mg/kg, ip), isoflurane + pizotifen, 8 mg/kg inactive PSD-95 mutant PDZ2 peptide (referred to as PDZ2MUT), 8 mg/kg active PSD-95 wild-type PDZ2 peptide (referred to as PDZ2WT), PDZ2MUT + pizotifen, and PDZ2WT + pizotifen. (**A**) Representative Western blot showing levels of phospho-CREB in crude hippocampal lysate in response to (**A**) gas and (**B**) peptide exposure in the presence or absence of 30 min treatment with pizotifen. Lower panel shows the densitometric analysis of p-CREB/Total CREB levels. Data were analyzed using 1-way ANOVA with Bonferroni’s multiple comparisons test. Data represent mean ± SD (n = 6 mice per group). ** *p* < 0.01 compared to control or PDZ2MUT or as indicated.

ERK-mediated CREB phosphorylation is critical for the induction of long-term memory [19]. Therefore, we investigated the effect of isoflurane or PDZ2WT peptides on CREB phosphorylation. We found a significant decrease in the phosphorylation of CREB in response to isoflurane. Moreover, injecting neonatal pups with 10 mg/Kg pizotifen for 30 min at the time of exposure prevented the decrease in phospho-CREB that occurred after isoflurane exposure in the hippocampus region of neonatal mice brains (Figure 2A). However, we did not see a significant loss of CREB phosphorylation in response to PDZ2WT peptide exposures (Figure 2B). The data were analyzed using one-way ANOVA, which showed that the levels of phosphorylated CREB relative to total CREB were significantly decreased in the isoflurane group compared to the control (*p* = 0.0048). No significant differences were observed between the PDZ2MUT peptide and PDZ2WT peptide group (*p* = 0.1461), between control + pizotifen and isoflurane + pizotifen (*p* = 0.1595), or between PDZ2MUT + pizotifen and PDZ2WT + pizotifen (*p* > 0.999). Six mice per group were used in the analysis.

### 2.3. Isoflurane or PDZ2WT Peptide-Induced Loss in Novel Object Recognition Memory in 5-Week-Old Male and Female Mice

**Figure 3:**
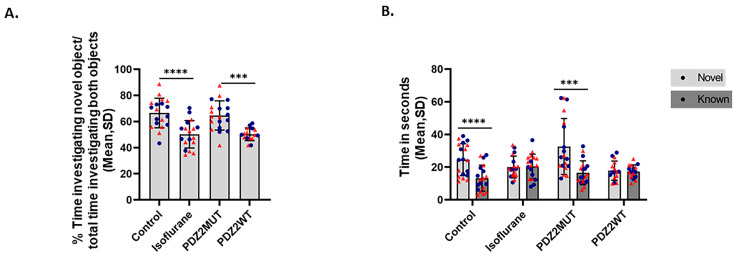
Neonatal exposure to isoflurane or wild-type PDZ2 peptide impairs novel object recognition memory at 5 weeks of age. Plots show percent of time mice spent investigating novel or known objects among experimental groups. One-week-old mice were exposed to (control conditions [50% oxygen: (control)], 1.5% isoflurane in 50% O_2_ (isoflurane), 8 mg/kg inactive PSD-95 mutant PDZ2 peptide (PDZ2MUT), and 8 mg/kg active PSD-95 wild-type PDZ2 peptide (PDZ2WT). Mice: control (n = 19), isoflurane (n = 18), PDZ2MUT (n = 19), and PDZ2WT (n = 16). n = number of mice. Data from individual animals are plotted and color-coded by gender (red = female and blue = male). Data are plotted as mean ± SD. (**A**) Left plot shows discrimination index as % time investigating novel object/total time investigating both objects X 100. Data were analyzed using one-way ANOVA with Bonferroni’s multiple comparisons test. (**B**) Right plot shows investigation time comparing novel versus known objects. Data were analyzed with paired *t*-tests. *p* < 0.050 was considered significant. *** *p* < 0.001, **** *p* < 0.0001 compared to control or PDZ2MUT or as indicated.

To determine whether the disruption of synaptic PSD95-PDZ2 interactions contributes to cognitive impairment after early anesthetic exposure and has long-lasting effects, we investigated the impacts of isoflurane and PDZ2WT peptides on nonspatial memory by assessing hippocampal-dependent object recognition in 5-week-old mice 4 weeks after exposure. The impairment in novel object recognition caused by isoflurane or PDZ2WT peptide exposures was calculated by measuring the discrimination index. The discrimination index = time investigating novel object over time investigating novel object plus known object × 100 (Figure 3A). The data were analyzed using one-way ANOVA, which indicated that the isoflurane-treated (n = 18) and PDZ2WT-treated (n = 16) mouse groups performed less well than the control (n = 19) and PDZ2MUT (n = 19) groups. The mean recognition index ± SD showed significant differences between control and isoflurane (*p* < 0.0001), as well as between PDZ2MUT and PDZ2WT (*p* = 0.0001). The values of n represent the number of mice in each group. All control mice (control and PDZ2MUT) were able to discriminate between novel and known objects revealed by significantly increased amounts of time investigating the novel object over the known object. In contrast, experimental mice (isoflurane and PDZ2WT) groups depict no significant increase in investigation time between the novel and known objects (Figure 3B). The time spent investigating the novel versus known object was analyzed using a paired *t*-test. The mean time (seconds) ± SD for novel versus known objects was determined in the control group (*p* < 0.0001) and the PDZ2MUT group (*p* = 0.0002). No significant differences were observed in the isoflurane group (*p* = 0.8609) or the PDZ2WT group (*p* = 0.5221).

### 2.4. Treatment with Pizotifen Prevented Isoflurane or PDZ2WT Peptide-Induced Loss in Novel Object Recognition Memory in 5-Week-Old Mice

**Figure 4:**
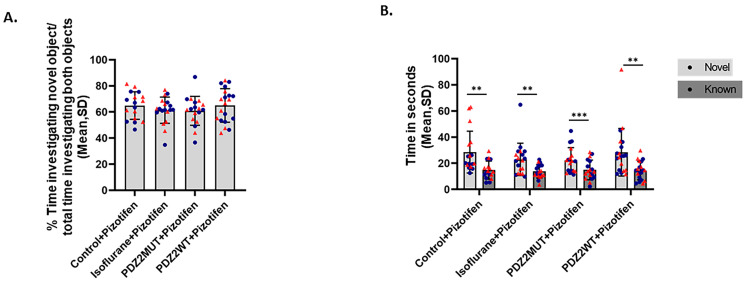
Pizotifen treatment during neonatal exposure to isoflurane or wild-type PDZ2 peptide prevents impairment in recognition memory at 5 weeks of age in both male and female mice. At 1 week of age, mice cohorts were exposed to oxygen (control conditions [50% oxygen: control]), 1.5% isoflurane in 50% O_2_ (isoflurane), 8 mg/kg inactive PSD-95 mutant PDZ2 peptide (PDZ2MUT), or 8 mg/kg active PSD-95 wild-type PDZ2 peptide (PDZ2WT). At the end of the exposure period (4 h after onset of exposure), all animals received 10 mg/kg of pizotifen (ip). Mice: control + pizotifen (n = 17), isoflurane + pizotifen (n = 19), PDZ2MUT + pizotifen (n = 18), and PDZ2WT + pizotifen (n = 20). n = number of mice. Data from individual animals are plotted and color-coded by gender (red = female and blue = male). (**A**) Left plot shows recognition index as % time investigating novel object/total time investigating both objects X 100. Data represent mean ± SD. Data were analyzed with 1-way ANOVA followed by Bonferroni’s multiple comparisons test. (**B**) Right plot shows investigation time comparing novel versus familiar objects. Data were analyzed with paired *t*-tests. *p* < 0.050 was considered significant. *** *p* < 0.001, ** *p* < 0.01, compared to as indicated.

We investigated whether pizotifen has any effect on cognitive impairment caused by isoflurane or PDZ2WT. We found that treatment with pizotifen prevented the impairment in novel object recognition caused by isoflurane and PDZ2WT peptides at 5 weeks of age. The discrimination index was evaluated in the presence of pizotifen. In the presence of pizotifen, mice were able to discriminate between novel and known objects (Figure 4A). Data were analyzed using 1-way ANOVA, which indicated no significant differences between control + pizotifen and isoflurane + pizotifen, (*p* = 0.6922) and PDZ2MUT + pizotifen and PDZ2WT + pizotifen, (*p* = 0.5306). In the presence of pizotifen, all cohorts were able to discriminate between the novel and familiar objects, as indicated by significantly increased amounts of time spent investigating the novel object compared to the familiar object (Figure 4B). The data were analyzed using a paired *t*-test, and the mean time (seconds) ± SD for novel versus familiar objects showed significant differences in all groups: control + pizotifen (n = 17, *p* = 0.0013), isoflurane + pizotifen (n = 19, *p* = 0.001), PDZ2MUT + pizotifen (n = 18, *p* < 0.001), and PDZ2WT + pizotifen (n = 20, *p* = 0.0019).

### 2.5. Neonatal Isoflurane or Wild-Type PDZ2 Peptide-Induced Loss in Mushroom Spine Density in 7-Week-Old Male- and Female-Sex Mice

We investigated whether the loss in the ERK activity in response to isoflurane or PDZ2WT peptide causes a decrease in mushroom spine density and if it is sex-specific. Neonatal mouse pups were exposed at 1 week of age to isoflurane or oxygen for 4 h. A separate cohort of both male and female mice were exposed to PDZ2MUT or PDZ2WT peptides. Six weeks after exposure (at 7 weeks of age), mice were euthanized, and the brains were dissected for rapid staining to visualize hippocampal dendritic spines within the superior blade of the dentate gyrus.

**Figure 5:**
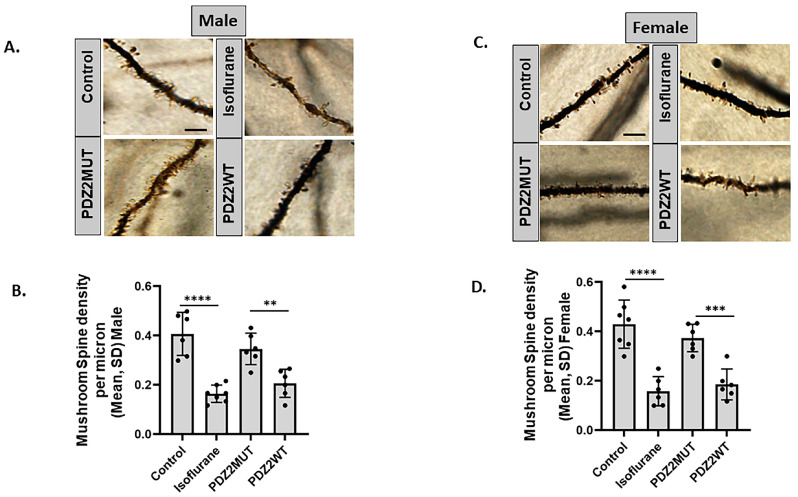
Neonatal exposure to isoflurane or wild-type PDZ2 peptide decreases hippocampal mushroom spine density in both male and female mice. At 1 week of age, mice in cohort 1 were exposed to oxygen (control), 1.5% isoflurane, PDZ2MUT, or PDZ2WT peptides. (**A**,**C**) Representative images showing dendritic segments and spines in (**A**) male and (**C**) female mice; scale bar 5 μm. (**B**,**D**) Summary plot represents the mushroom spine density among exposed groups assessed at seven weeks in (**B**) male and (**D**) female mice. Data represent mean ± SD. Male mice: control (n = 6), isoflurane (n = 7), PDZ2MUT (n = 6), and PDZ2WT (n = 6). Female mice: control (n = 7), isoflurane (n = 6), PDZ2MUT (n = 6), and PDZ2WT (n = 6). n = number of mice. Data were analyzed with 1-way ANOVA followed by Bonferroni’s multiple comparisons test. *p* < 0.050 was considered significant. ** *p* < 0.01, *** *p* < 0.001, **** *p* < 0.0001 compared to control or PDZ2MUT or as indicated.

We found that isoflurane or PDZ2WT peptide had a significant effect on the number of mushroom spines in both male and female mice. Mushroom spine density was quantified along the dendritic segments distal to the first and second branch points (Figure 5A–D). Data were analyzed separately for male and female mice using one-way ANOVA. Both isoflurane and PDZ2WT peptide significantly affected the number of mushroom spines at 7 weeks of age. For male mice, the mean spine density (spines/μm) ± SD showed significant differences between control (n = 6) and isoflurane (n = 7) (*p* < 0.0001) and between PDZ2MUT (n = 6) and PDZ2WT (n = 6) (*p* = 0.0018). In female mice, the mean spine density (spines/μm) ± SD revealed significant differences between control (n =7) and isoflurane (n = 6) (*p* < 0.0001) and between PDZ2MUT (n = 6) and PDZ2WT (n = 6) (*p* = 0.0004).

### 2.6. Pizotifen Prevented Neonatal Isoflurane or PDZ2WT Peptide-Induced Loss in Mushroom Spine Density in 7-Week-Old Male and Female Mice

**Figure 6:**
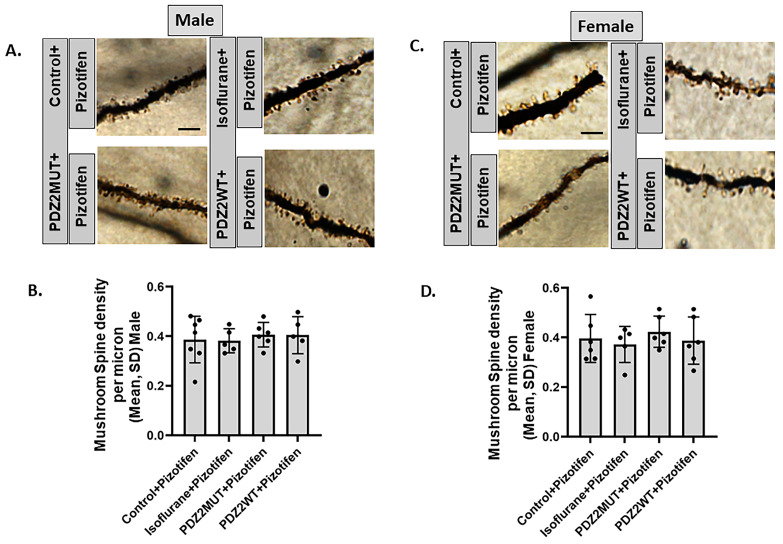
Treatment with pizotifen prevents the decrease in mushroom spine density in seven-week-old male and female mice induced by neonatal exposure to isoflurane or wild-type PDZ2 peptide. Density of mushroom spines among exposed groups was evaluated at seven weeks. At 1 week of age, mice cohorts were exposed to oxygen (control), isoflurane, PDZ2MUT, or PDZ2WT. At the end of the exposure period (4 h after onset of exposure), all animals received 10 mg/kg of pizotifen (ip). (**A**,**C**) Representative images showing dendritic segments and spines in (**A**) male and (**C**) female mice; scale bar 5 μm. (**B**,**D**) Summary plots represent the mushroom spine density among exposed groups assessed at seven weeks in (**B**) male and (**D**) female mice. Data are plotted as mean ± SD. Male mice: control + pizotifen (n = 7), isoflurane + pizotifen (n = 5), PDZ2MUT + pizotifen (n = 6), and PDZ2WT + pizotifen (n = 5). Female mice: control + pizotifen (n =6), isoflurane+ pizotifen (n = 5), PDZ2MUT + pizotifen (n = 6), and PDZ2WT + pizotifen (n =6). n = number of animals. Data were analyzed with 1-way ANOVA followed by Bonferroni’s multiple comparisons test. *p* < 0.050 was considered significant.

We investigated if preventing the loss in ERK phosphorylation by using pizotifen is also sufficient to prevent the loss in mushroom spine density in response to isoflurane or PDZ2WT peptides. Neonatal mouse pups were exposed at 1 week of age to isoflurane or oxygen for 4 h. A separate cohort of both male and female animals were injected with PDZ2MUT or PDZ2WT peptides in the presence and absence of pizotifen. Mice were euthanized, and the brains were dissected 6 weeks later at 7 weeks of age for rapid staining to visualize hippocampal dendritic spines within the superior blade of the dentate gyrus. We found that pizotifen prevented the loss of mushroom spine density in response to isoflurane or PDZ2WT peptides in both male and female mice. Mushroom spines were quantified along the dendritic segments distal to the first and second branch points (Figure 6A,C). Data were analyzed using one-way ANOVA. For male mice, the mean spine density (spines/μm) ± SD showed no significant differences between control + pizotifen (n = 7) and isoflurane + pizotifen (n = 5) (*p* > 0.999) or between PDZ2MUT + pizotifen (n = 6) and PDZ2WT + pizotifen (n = 5) (*p* > 0.999). In female mice, there were no significant differences between control + pizotifen (n = 6) and isoflurane + pizotifen (n = 5) (*p* > 0.999) or between PDZ2MUT + pizotifen (n = 6) and PDZ2WT + pizotifen (n = 6) (*p* = 0.932).

## 3. Discussion

**Figure 7:**
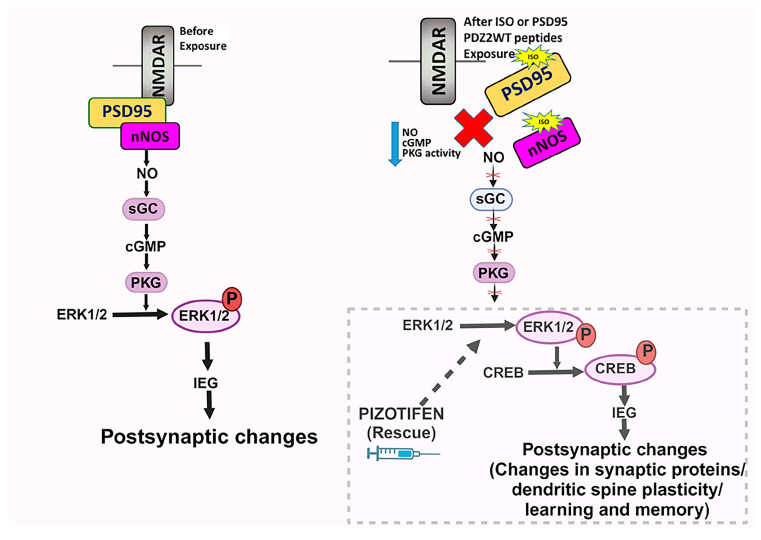
Schematic representation illustrates the underlying mechanisms in response to the dissociation of the NMDAR-PSD95-nNOS interaction induced by isoflurane or PDZ2WT peptide. Basal conditions: NMDAR-PSD-95/93-nNOS complexes help Ca^2+^ influx through NMDAR on the postsynaptic membrane to activate nNOS. The NO generated is required for neuronal plasticity. It acts predominantly via cGMP and protein kinase-G (PKG) for the activation of kinase pathways. After exposure: disruption of NMDAR-PSD-95/93-nNOS complexes. This disruption can reduce the efficacy with which calcium ions activate nNOS. NO is also required for ERK activation and for the full expression of plasticity-related proteins, an effect primarily mediated by cGMP and PKG [13,21]. However, using pizotifen may activate the ERK pathway and preserve the loss in spine plasticity and cognitive dysfunction that occurred in response to impaired nNOS signaling. Graphical abstract is Created with BioRender.com.

To achieve a more comprehensive understanding of the mechanisms underlying anesthesia-induced neurotoxicity and long-term cognitive abnormalities, this study investigated downstream signaling components as specific molecular targets of anesthesia (Figure 7). In previous studies, we demonstrated that inhalational anesthetics at clinically relevant concentrations disrupt synaptic PDZ2 domain-mediated protein–protein interactions between PSD-95 or PSD-93 and the NMDA receptor NR2 subunits or neuronal nitric oxide synthase (nNOS) [13,17,20]. Additionally, treatment with an NO donor or cGMP analog during exposure attenuated the loss of early dendritic spines, mature synapses, and the diminution in cyclic GMP-dependent protein kinase (PKG) activity in response to isoflurane or PDZ2WT peptide in primary neuronal cultures [13]. Our earlier studies indicated that the dysregulation of NMDAR NR2-PSD-95-PDZ2-nNOS ternary complexes following neonatal anesthetic exposure perturbs the downstream NO/sGC/cGMP-mediated PKG signaling pathway. Using the soluble guanylyl cyclase (sGC) activator YC-1 during early isoflurane exposure in mice prevented the loss of object recognition memory at 5 weeks of age [13].

In this study, we demonstrated that exposure to isoflurane or PDZ2WT peptides in neonates impairs the extracellular signal-regulated kinase (ERK)-cAMP response element-binding protein (CREB) pathway, resulting in the loss of learning and memory functions. Extracellular signal-regulated kinases (ERKs) are abundantly expressed and crucial for relaying extracellular signals into intracellular responses. ERKs are part of the mitogen-activated protein kinase (MAPK) family and play a critical role in communicating surface receptor signals to the nucleus [22,23]. ERKs also contribute to long-term potentiation (LTP) and spine structural plasticity, synaptic transmission, regulation of neuronal gene expression, and protein synthesis, leading to structural synaptic changes associated with learning and memory [23,24,25,26,27].

Our study found that exposure to isoflurane or PDZ2WT peptides for 4 h at postnatal day 7 (PND7) decreased ERK phosphorylation, while injecting mice with pizotifen for 30 min after exposure prevented the loss of ERK activation in the hippocampal tissue (Figure 1A,B). This aligns with other studies showing that anesthesia exposure can rapidly deteriorate the phosphorylation status of ERK in the brain cortex [28]. Another study suggested that the transient blockade of ERK phosphorylation during critical developmental periods causes autistic phenotypes in adult mice [29]. Additionally, we observed a loss in CREB phosphorylation in response to isoflurane, which was prevented in the presence of pizotifen (Figure 2A). However, we did not see a significant decrease in CREB phosphorylation in response to PDZ2WT peptide injection (Figure 2B), potentially because ERK1/2 can also directly phosphorylate synaptic proteins [30]. Previous studies indicated that ERK1/2 activity in the brain is primarily involved in long-term memory formation and expression [25]. ERK1/2 signaling is critical for memory processing, and relearning also requires ERK1/2 activity in certain brain regions [23,31,32,33]. Pizotifen treatment activates ERK1/2 signaling and provides neuroprotection in models of Huntington’s disease [34]. We demonstrated that injecting pizotifen at the time of isoflurane or PDZ2WT peptide exposure in neonatal mice pups prevented impairment in object recognition memory at 5 weeks of age in both male and female mice. The role of ERK in discrete memory stages highlights its importance as a core element in memory processing and a potential target for treating memory impairments associated with neurological disorders [23,31,32,33].

We investigated the critical role of ERK activation in dendritic spine plasticity in response to isoflurane and PDZ2WT peptides in both male and female mice. Previous studies have reported alterations in spine plasticity at PND21 and PND49 after exposure at PND7 in mice [17,20]. Evidence suggests sex-specific features of spine densities in the hippocampus [35,36]. Our results showed that neonatal exposure to isoflurane or PDZ2WT peptide decreased hippocampal mushroom spine density in both male and female mice at 7 weeks of age.

We also investigated whether using pizotifen could prevent this loss in mushroom spines. ERK activation supports dendritic spine plasticity by regulating synaptic proteins and promoting the formation of new dendritic spines, thereby maintaining LTP [19,24,37]. ERK is a key cellular component for the formation, retrieval, reconsolidation, and persistence of memory [38,39]. Our findings demonstrated that injecting pizotifen at the time of isoflurane or PDZ2WT peptide exposure in neonatal mice pups prevented the loss in mushroom dendritic spines at 7 weeks of age, supporting the role of the ERK pathway in mediating cellular changes associated with cognitive impairment.

Our present findings demonstrated that the dysregulation of NMDAR NR2-PSD-95-PDZ2-nNOS ternary complexes upstream after early anesthetic exposure impaired the downstream ERK pathway. Previous studies have shown that surgery and anesthesia increase brain-derived natriuretic peptide, which inhibits ERK-CREB signaling to decrease GDNF in the brain. The decrease in GDNF may also lead to inflammation and a reduction in synaptic proteins [40].

In conclusion, our results suggest that a single 4 h exposure of neonatal mice to 1.5% isoflurane or targeted disruption of postsynaptic density 95-PDZ2-mediated protein interactions using PDZ2WT peptide resulted in a persistent decrease in mushroom spine density at 7 weeks of age in both male and female mice, along with impairments in hippocampus-dependent learning and memory at 5 weeks of age. The loss in mushroom spine density can be attenuated by the introduction of pizotifen, suggesting the involvement of ERK as a crucial target component within the PKG signaling pathway in these processes. These findings highlight the complex interplay between the NO-cGMP-PKG and ERK/CREB signaling pathways in maintaining synaptic and spine plasticity, which are essential for cognitive functions.

The major limitations of the present study include the lack of investigation into the effect of pizotifen on the activation of the ERK pathway, which is crucial for the induction and maintenance of long-term potentiation (LTP), an essential mechanism for learning and memory. While we have previously demonstrated the prevention of LTP deficits using a nitric oxide (NO) donor upstream in a similar pathway, particularly in mediating cognitive impairment induced by isoflurane or PDZ2WT, the effects of pizotifen in this context remain unexplored. Additionally, the current study does not address the impact of pizotifen on the phosphorylation of synaptic proteins such as synapsin or spinophilin. Although we have shown that pizotifen prevents the loss of mushroom spines in response to isoflurane and PDZ2WT exposures, further investigation is needed to explore these molecular mechanisms in more detail.

## 4. Materials and Methods

### 4.1. Material

RIPA buffer, Pierce™ BCA Protein Assay Kit, Halt™ Protease and Phosphatase Inhibitor Cocktail, and Protein ladder were from Thermo Fisher Scientific (Waltham, MA, USA). The purified fusion peptides, active Tat-PSD-95 wild-type PDZ2 peptide (referred to as PDZ2WT peptides), and inactive Tat-PSD-95 mutant PDZ2 peptide (referred to as PDZ2MUT peptides) were purchased from Creative BioMart (Shirley, NY, USA) and used as previously described [13,15]. The 10× Tris-buffered saline (TBS; 1706435), 4× Laemmli sample buffer, 4–20% Criterion™ TGX™ Precast Midi Protein Gels, and 10× Tris/Glycine/SDS buffer (1610732) were purchased from Bio-Rad (Hercules, CA, USA). FD Rapid GolgiStain™ Kit was purchased from FD NeuroTechnologies, Inc. (Columbia, MD, USA). Pizotifen (20765) was purchased from Cayman Chemical (Ann Arbor, MI, USA).

### 4.2. Animals

This study was executed with approval from the Animal Care and Use Committee at Johns Hopkins University and was consistent with the National Institutes of Health’s Guide for the Care and Use of Laboratory Animals. Wild-type C57BL/6 mice of both sexes purchased from (The Jackson Laboratories, Bar Harbor, ME, USA) were maintained on a 12:12 h light:dark cycle, on corncob bedding (7097 Teklad, inotiv, Madison, WI, USA), with ad libitum access to chlorinated reverse-osmosis water and rodent diet (2018sx Teklad global 18% protein, inotiv, Madison, WI, USA) and environmental enhancement in the form of Nestlets (Ancare, Bellmore, NY, USA). Water and food were available ad libitum until mice were transported to the laboratory nearly 1 h before the experiments.

Postnatal day 7 mice were randomly assigned to control and treatment groups for in vivo experiments. Control mice at postnatal day 7 were exposed to 50% O_2_ (oxygen control group) or 8 mg/kg inactive postsynaptic density-95 mutant-type PDZ2 peptide (referred to as PDZ2MUT). Experimental group mice cohorts were exposed to 1.5% isoflurane in 50% O_2_ for 4 h or injected with 8 mg/kg active postsynaptic density-95 wild-type PDZ2 peptide (referred to as PDZ2WT). A subset of these mice also received 10 mg/kg of pizotifen injections.

All the mice were placed in clear plastic cones, and body temperature was maintained by a heating pad set to 37 °C. Four hours after gas exposures or peptide injections, mice were decapitated, and crude hippocampal tissue was dissected from the brain for biochemistry experiments. For the behavior and dendritic spine analysis experiments, both the male and female mice at postnatal day 7 were randomly assigned to 8 groups: (1) oxygen control, (2) oxygen control + pizotifen, (3) isoflurane, (4) isoflurane + pizotifen, (5) PSD95-MUTPDZ2 peptide, (6) PSD95-MUTPDZ2 peptide + pizotifen, (7) PSD95-WTPDZ2 peptide, and (8) PSD95-WTPDZ2 peptide + pizotifen.

### 4.3. Western Blotting

After all the exposures, mice were euthanized by decapitation, and hippocampal tissues were dissected from the brains using a dissection microscope. Total protein was extracted using RIPA buffer (Thermo Scientific, Waltham, MA, USA) according to the manufacturer’s instructions. The hippocampal tissue was dissected and homogenized in a bead homogenizer in the presence of ice-cold RIPA buffer (0.025 M Tris, 0.15 M NaCl, 0.001 M EDTA, 1% NP-40, 5% glycerol, pH 7.4) supplemented with a protease and phosphatase inhibitor cocktail. The crude homogenates were centrifuged at 16,000× *g* for 20 min at 4 °C. The lysates were collected, and protein concentration was estimated with the Pierce™ BCA Protein Assay Kit, according to the manufacturer’s instructions. The protein was then resolved by SDS-PAGE and immunoblotted through Western blotting. Proteins from the gel were transferred to polyvinylidene difluoride membranes purchased from Millipore (Burlington, MA, USA).

The membranes were blocked in tris-buffered saline + 0.1% Tween-20 containing 5% nonfat milk for 1 h at room temperature and then incubated with primary antibodies to phospho p44/p42 MAPK (Erk1/2) (Thr202/Tyr204) Rabbit mAb (cat # 4370) (1:1000) or p44/42 MAPK (Erk1/2) Rabbit mAb (cat # 4695) (1:1000) procured from Cell Signal Technology, Danvers, MA, USA or Phospho-CREB Recombinant Rabbit Monoclonal Antibody (cat # 700129) (1:1000) or CREB Monoclonal Antibody (cat # MA1-083) (1:1000) procured from Thermo Scientific, Waltham, MA, in tris-buffered saline + 0.1% Tween-20 containing 5% nonfat milk overnight at 4 °C. Membranes were washed 3 times for 10 min each in tris-buffered saline + 0.1% Tween-20 and then incubated for 2 h with horseradish peroxidase-conjugated anti-rabbit (cat # 7074S) or anti-mouse immunoglobulin (cat # 7076S) procured from Cell Signal Technology and used at a dilution of 1:5000. Finally, the membranes were washed to remove excess secondary antibody. The 10× Tris-buffered saline (TBS; 1706435), 4× Laemmli sample buffer, Criterion™ TGX™ Precast Midi Protein Gels, and 10× Tris/Glycine/SDS buffer (1610732) were purchased from Bio-Rad (Hercules, CA, USA).

Proteins were visualized with enhanced chemiluminescence (Amersham, Piscataway, NJ, USA), and images were captured with the ChemiDoc Imaging System (Bio-Rad, Hercules, CA, USA). Immunoblots were quantified using Bio-Rad Quantity One image analysis software version 4.6.2 (basic).

### 4.4. Novel Object Recognition

The novel object recognition procedure was performed as reported by previous studies [13,17,41,42,43] and was used to evaluate nonspatial hippocampal memory [41,42]. It comprised 2 sessions. First was a familiarization session during which mice were allowed to freely discover 2 similar objects within an opaque box (40 cm W × 40 cm L × 34 cm H) for 10 min. This was followed by a second session after 2 h, which is a test session in which one of the objects is replaced by a novel or unacquainted object, and mice were allowed to explore for 5 min. Mice distinctively prefer to explore the novel object relative to the familiar one, and a preference for the novel object demonstrates intact memory for the familiar object. Object investigation time was determined by the amount of time the mouse spent in the zone immediately surrounding the object. Only mice that investigated any object for at least 10 s (criterion) were taken into consideration. During the testing phase (n = 9), mice that did not meet the criterion were not included in the analysis. Test analysis data were recorded with a video camera, and time spent with each object was analyzed by ANY maze software version 7.43 (Stoelting, Wood Dale, IL, USA).

### 4.5. Golgi Staining, Microscopy, and Spine Reconstruction

Golgi staining was performed following previously published papers [17,20]. At 7 weeks of age, mice were deeply anesthetized and perfused transcardially with a brief flush of 0.01 M phosphate-buffered saline (pH 7.4), followed by 50 mL of 4% paraformaldehyde in 0.1 M phosphate buffer (pH 7.4). After the perfusion, the brains were removed and stained using FD Rapid Golgi Stain Kit (FD Neuro Technologies, Inc., Columbia, MD, USA) as per the manufacturer’s instructions. Brain tissues were immersed in AB impregnation solution at room temperature in the dark for 2 weeks. Impregnation solution was replaced after the first overnight on the next day. Tissue was transferred to solution C for 72 h. Brain tissues were embedded in tissue freezing medium and stored at −80 °C. Then, 60-micron sections were cut on a cryostat at −20 °C, mounted onto gelatin-coated slides, and air-dried overnight. Slides were rinsed in Milli-Q water, developed in the working solution DE for 10 min, rinsed, dehydrated in ethanol, cleared in xylene, and mounted with Permount^®^ procured from Fisher Scientific, Waltham, MA, USA.

Two different imaging fields per mouse, each containing at least 3 unique dendritic segments that contained dorsal hippocampus, were imaged (6 segments total per mouse). The 6 segments were averaged per mouse to contribute one datapoint per mouse. Dentate granule cells were identified by their location within the dentate gyrus and their distinct morphology, as shown previously [17,20]. Spines along the secondary and tertiary dendrites of these neurons were selected for analysis. Z-stacks of Golgi-stained dendrites (optical section thickness = 0.3 μm, i.e., 50–100 images per stack) were taken at 630× magnification, with pixel size (px) 1920 × 1440 on a Leica SPE confocal microscope (Wetzlar, Germany).

Spine analysis was performed as described in Risher et al. (2014) [44] using the freely available RECONSTRUCT (version 1.1.0.0) software [45]. Objective classification of dendritic spines was done from the Golgi-stained tissue described above. This approach utilizes the distinct geometric characteristics of spines as the basis for their categorization. Spine head width and neck length measurements were obtained. Spines having (width > 0.6 μm) were marked for mushroom spines. Individuals performing imaging and reconstruction were naïve to the presumed outcome (so essentially blinded).

### 4.6. Statistical Analysis

All statistical analyses were carried out using GraphPad Prism version 10.3 (GraphPad Inc., San Diego, CA, USA). All data were tested for normality and then presented as mean ± standard deviation. Paired *t*-test or 1-way ANOVA were used for statistical analysis. For multiple testing corrections, Bonferroni’s post hoc test was used when data between selected groups were compared. Statistical significance was set at *p* < 0.05. Sample sizes were chosen based on preliminary data and/or previously published studies [13,20].

## Data Availability

Data is contained within the article.
